# Islands of runs of homozygosity indicate selection signatures in *Ovis aries* 6 (OAR6) of French dairy sheep

**DOI:** 10.3168/jdsc.2020-0011

**Published:** 2021-03-26

**Authors:** S.T. Rodríguez-Ramilo, A. Reverter, A. Legarra

**Affiliations:** 1 GenPhySE, Université de Toulouse, INRAE, ENVT, F-31326, Castanet Tolosan, France; 2 CSIRO Agriculture & Food, Brisbane, QLD 4067, Australia

## Abstract

Runs of homozygosity (ROH) are contiguous homozygous segments of the genome where the haplotypes inherited from each parent are identical. The occurrence of ROH is not randomly distributed across the genome, and ROH islands across many animals may be the result of selective pressure. The objective of this study was to demonstrate that the presence of ROH islands may be indicative of selection signatures in French dairy sheep breeds and subpopulations. The data set available included animals (artificial insemination males) from various breeds and subpopulations: Basco-Béarnaise breed (321 individuals), Manech Tête Noire breed (329 individuals), Manech Tête Rousse breed (1,906 individuals), Lacaune Confederation subpopulation (3,030 individuals), and Lacaune Ovitest subpopulation (3,114 individuals). Animals were genotyped with the Illumina OvineSNP50 BeadChip. After applying filtering criteria, the genomic data included 38,287 autosomal SNP distributed across 26 chromosomes and 8,700 individuals. One island of ROH was detected on OAR6 in the same genomic position across animals (between 30 and 40 Mb). Global Wright's differentiation coefficients for 2 SNP within this ROH island were high (0.67–0.68). The linkage disequilibrium between both SNP was also elevated (0.98). The divergence in allele frequencies in those SNP grouped Basco-Béarnaise, Manech Tête Noire, and Manech Tête Rousse breeds in one cluster and Lacaune Confederation and Lacaune Ovitest subpopulations in another cluster. The closest candidate genes are *NCAPG* and *LCORL*, which have been reported to be under positive selection and suggested to control weight and height in sheep. The preliminary identification of ROH suggests the presence of selection. However, for the identification of potential candidate genes, ROH detection should be combined with other approaches to improve mapping accuracy.

## Highlights

• The presence of runs of homozygosity is not randomly distributed across the genome.
• Islands of runs of homozygosity may be the result of selection pressure.
• Concordance existed between islands of runs of homozygosity and selection signatures on OAR6.
• Candidate genes *NCAPG* and *LCORL* on OAR6 have agricultural and adaptive importance.

## Graphical Abstract

**Figure:**
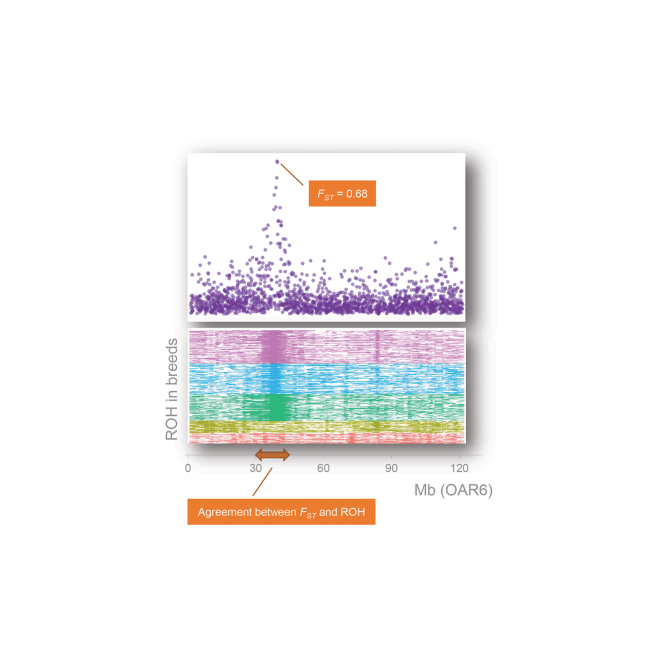
**Summary:** The occurrence of runs of homozygosity (ROH) is not randomly distributed across the genome, and ROH islands across many animals may be the result of selective pressure. The objective of this study was to demonstrate that the presence of ROH islands may be indicative of selection signatures in French dairy sheep breeds and subpopulations. One island of ROH was detected on OAR6 in the same genomic position across animals (between 30 and 40 Mb). Global Wright's differentiation coefficients (F_ST_) for 2 SNP (OAR6_41583796.1 and OAR6_41709987.1) within this ROH island were high (0.67–0.68). ROH and single SNP FST analyses agreed that selection signatures exist around both SNP that were located near the *NCAPG* and *LCORL* genes.

Sheep, one of the first domesticated species, have a manageable size and the ability to adapt to different climates and diets with poor nutrition (Zeder, 2008). From their domestication, sheep spread worldwide, and natural and artificial selection shaped a large variety of breeds with different morphology, coat colors, and specialized production in meat, milk, or wool. These selection mechanisms have left signatures in domestic animal genomes, such as decreased genetic diversity and long haplotypes (Rochus et al., 2018).

Different methods varying in the underlying assumptions have been proposed to detect selection signatures, such as the genome-wide differentiation coefficient (**F_ST_**; Weir and Cockerham, 1984), extended haplotypes of homozygosity (Sabeti et al., 2002), and integrated haplotype homozygosity score (**iHS**; Voight et al., 2006). Genome-wide scans for detecting selection signatures have been successfully applied to domestic animals based on these approaches.

The availability of large numbers of SNP has made these markers appropriate to distinguish old (short runs) from new (long runs) identical by descent segments (Howrigan et al., 2011). Accordingly, SNP can be used for the detection of genomic regions where an increase in homozygosity has occurred. Although the presence of copy number variation or large distance between SNP can interfere with the detection of regions of homozygosity (Nandolo et al., 2018), the identification of runs of homozygosity (**ROH**) is a potential alternative to detect signatures of selection. The occurrence of ROH is heterogeneous along the genome due to the stochastic nature of the recombination events. Consequently, the detection of ROH islands may be indicative of selective pressure. Runs of homozygosity can provide a better understanding about the population selection history, supporting the discovery of genes or genomic regions under selective pressure (Zhang et al., 2015).

Selection of French dairy sheep has been implemented for each local breed and subpopulation separately. There are 3 major regions for sheep milk production. The first is around the Roquefort (Aveyron) area, with the Lacaune breed, whose elite population (breeding flocks) consists of 2 subpopulations, Lacaune Confederation (**LACCon**) and Lacaune Ovitest (**LACOvi**), which split around 1975 and proceed in parallel selection schemes. In the second region, the Western Pyrenees mountains, there are 3 breeds: Manech Tête Rousse (**MTR**), Manech Tête Noire (**MTN**), and Basco-Béarnaise (**BB**). The third region is Corse, but Corsican sheep were not included in the present study. All of the breeds in this study (LACCon, LACOvi, MTR, MTN, and BB) have been selected for production (milk yield and composition) and functional (resistance to mastitis, udder morphology) traits with varying intensities. The objective of this study was to demonstrate that the presence of ROH islands may be indicative of selection signatures in French dairy sheep breeds and subpopulations.

Rams were genotyped with the OvineSNP50 BeadChip (Illumina Inc.). The number of genotyped animals was 321, 329, 1,906, 3,030, and 3,114 for BB, MTN, MTR, LACCon, and LACOvi, respectively. These are AI rams representing several years of birth (10–15) and overlapping generations, and they illustrate the genetic variability of each breed. The SNP quality control included the absence of parent–offspring Mendelian segregation incompatibilities (<3%), SNP call rate >97%, and large deviations from Hardy-Weinberg equilibrium (SNP with *P* < 10^−6^ were discarded). The final data set included 8,700 genotyped individuals and 38,287 autosomal SNP. Markers were positioned using the sheep (*Ovis aries*) genome assembly 3.1 (OAR 3.1; https://www.ncbi.nlm.nih.gov/assembly/GCF_000298735.1/). See Legarra et al. (2014) for more details.

Plink software version 1.9 (Chang et al., 2015) was used to calculate the genetic differentiation coefficient F_ST_ (Weir and Cockerham, 1984) between subpopulations and breeds with the option –fst. Significance level was evaluated using Lewontin and Krakauer (1973) statistical test (Bonhomme et al., 2010) and applying a Bonferroni correction using the total number of markers.

The rehh R package (Gautier and Vitalis, 2012) with the default values was used to calculate iHS, where an extreme score (>2 or <−2) provides a strong signal of selection (Voight et al., 2006), indicating the most extreme 5% of iHS values. The software detectRUNS (Biscarini et al., 2018) was used to detect ROH with a sliding window–based method and the following criteria: a minimum length of 250 kb, a minimum of 20 homozygous SNP, a maximum distance of 1 Mb allowed between 2 consecutive homozygous SNP in a run, a minimum density of 1 SNP per 10 kb, and no missing or heterozygous genotypes allowed.

The correlation between the number of times an SNP is included within an ROH and F_ST_ (or iHS) was calculated for all SNP in each chromosome. The average genetic differentiation coefficient across breeds and subpopulations was 0.0576 ± 0.0003. The genome-wide distribution of global differentiation coefficient for each SNP revealed that the highest selection signal was detected on OAR6 (Figure 1a). The 2 highest ranked SNP (*OAR6_41583796.1*, F_ST_ = 0.675, *P* = 4.03 × 10^−9^; and *OAR6_41709987.1*, F_ST_ = 0.670, *P* = 4.01 × 10^−9^) were located at 37,423,374 and 37,542,319 bp, respectively. Three other significant SNP were also detected on OAR6 near the 2 highest ranked SNP at 36,205,101, 36,972,847, and 37,318,895 bp). In addition, 6 isolated significant SNP were detected on OAR3 (2 SNP; 61,548,300 and 61,577,699 bp), OAR11 (1 SNP; 40,118,817 bp), OAR13 (2 SNP; 46,841,439 and 62,932,554 bp), and OAR16 (1 SNP; 33,219,172 bp). The F_ST_ values for the remaining autosomes were not as high as the F_ST_ values observed for the 2 highest ranked SNP on OAR6. Results obtained for the iHS derived statistic are shown in Figure 1b. A total of 1,735 significant SNP were detected in all chromosomes for the iHS statistic. Agreement between significant SNP for F_ST_ and iHS was detected only for 1 SNP on OAR13 on 46,841,439 bp. A total of 95 significant SNP for iHS were observed in OAR6. At least 2 significant SNP for iHS on OAR6 (*OAR6_39124095.1* and *OAR6_42392875.1*) were close (35,063,421 and 38,193,916 bp, respectively) to the highest significant SNP for F_ST_.

**Figure 1:**
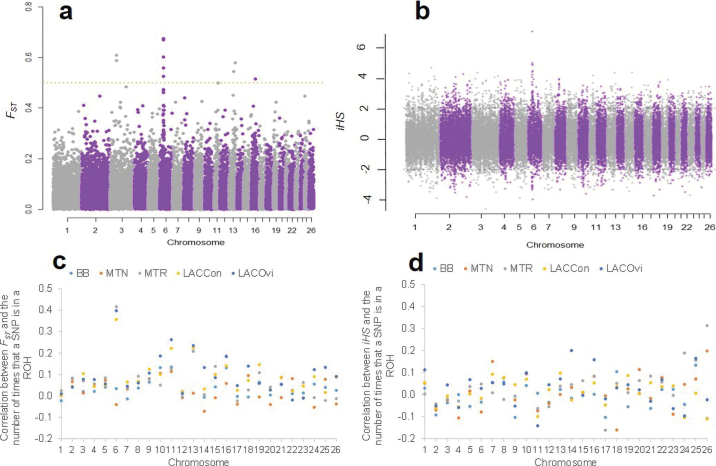
(a) Genome-wide differentiation coefficient (F_ST_) across chromosomes. Orange dashed line indicates the significance level after Bonferroni correction. (b) Integrated haplotype homozygosity score (iHS). Black dashed lines indicate the iHS threshold (>2 or <−2). (c) Correlation between F_ST_ and the number of times that an SNP is in an ROH. (d) Correlation between iHS and the number of times that a SNP is in an ROH. BB = Basco-Béarnaise; MTN = Manech Tête Noire; MTR = Manech Tête Rousse; LACCon = Lacaune Confederation; LACOvi = Lacaune Ovitest.

The highest correlation between F_ST_ and the number of times that an SNP was in an ROH was observed in OAR6 for LACOvi and LACCon subpopulations and MTR breed ranging between 0.36 and 0.42 (Figure 1c). However, the highest correlation (0.31) between iHS and the number of times that an SNP was in an ROH was observed in OAR26 for the MTR breed (Figure 1d). The correlation between iHS and the number of times that an SNP was in an ROH does not support the OAR6 signal observed in Figure 1c. This can be related to the fact that F_ST_ and iHS have identified different significant SNP within OAR6.

The F_ST_ values for SNP *OAR6_41583796.1* and *OAR6_41709987.1* between pairs of breeds and subpopulations are shown in Table 1. The highest F_ST_ values were observed between Western Pyrenees breeds and Lacaune subpopulations.

**Table 1:** Differentiation coefficient between pairs of breeds and subpopulations for SNP *OAR6_41583796.1* (above the diagonal) and *OAR6_4170998*7*.1* (below the diagonal)

Breed^1^	BB	MTN	MTR	LACCon	LACOvi
BB		0.077	0.131	0.652	0.777
MTN	0.076		0.403	0.481	0.639
MTR	0.129	0.397		0.784	0.860
LACCon	0.649	0.479	0.782		0.019
LACOvi	0.771	0.633	0.856	0.018	

1 BB = Basco-Béarnaise; MTN = Manech Tête Noire; MTR = Manech Tête Rousse; LACCon = Lacaune Confederation; LACOvi = Lacaune Ovitest.

The allele frequencies observed for both SNP also diverged for the same allele between Western Pyrenees breeds (BB, MTN, and MTR) and Lacaune subpopulations (LACCon and LACOvi). For SNP *OAR6_41583796.1*, the allele frequency ranged between 0.62 and 0.95 in Western Pyrenees breeds and between 0.07 and 0.13 in Lacaune subpopulations for the same allele. In other words, they were nearly fixed for opposite alleles. Similar results were observed for SNP *OAR6_41709987.1*. In addition, both SNP were in almost complete linkage disequilibrium (calculated from allele frequencies, R^2^ = 0.98).

Figure 2a shows the ROH island detected for each animal on OAR6. The frequency of ROH was high between 30 and 40 Mb in LACOvi and LACCon subpopulations and the MTR breed. This ROH island included both SNP *OAR6_41583796.1* and *OAR6_41709987.1* (the highest ranked SNP for F_ST_). The ROH island detected on OAR6 involves a large interval compared with the reduced interval obtained from the 2 most significant SNP in the F_ST_ analysis. This suggests that the F_ST_ approach is more precise than ROH methodology in terms of mapping accuracy. However, the gene mapping accuracy of ROH is affected by the criteria used to define an ROH, which in turn depends on the available genotype SNP density. In any case, ROH signals suggest that the hitchhiking effect involves a large region of homozygosity around the potential candidate genes. Some agreement between ROH islands and selection signatures has been previously reported in the literature (e.g., Purfield et al., 2017). Other regions of high percentage of time each SNP falls inside an ROH were also detected in OAR2, OAR4, and OAR10 (Figure 2b), but no concordance between those homozygous regions and strong differentiation coefficient was observed. This lack of agreement could be attributed to demography and recombination events giving rise to ROH (Bosse et al., 2012).

**Figure 2:**
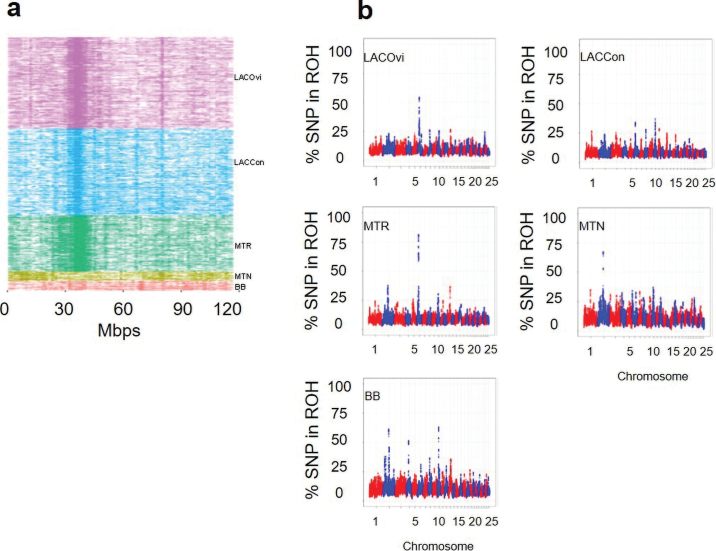
(a) Runs of homozygosity detected for each animal on OAR6. Mbps = megabases. (b) Percentage of times each SNP falls inside an ROH. BB = Basco-Béarnaise; MTN = Manech Tête Noire; MTR = Manech Tête Rousse; LACCon = Lacaune Confederation; LACOvi = Lacaune Ovitest.

The selection signal identified on OAR6 has been previously reported in the literature (e.g., Al-Mamun et al., 2015; Manunza et al., 2016; Gutiérrez-Gil et al., 2017). Fariello et al. (2014) found on OAR6 between 33.22 and 41.02 Mb a signal of selection, and the candidate genes associated with this position were *ABCG2* (located in OAR6 between 36,514,210 and 36,556,824 bp on the OAR 3.1 reference genome), *NCAPG* (located in OAR6 between 37,256,548 and 37,333,851 bp on the OAR 3.1 reference genome), and *LCORL* (located in OAR6 between 37,365,236 and 37,452,332 bp on the OAR 3.1 reference genome). *ABCG2* has been associated with a strong QTL for milk production in cattle, and *NCAPG* and *LCORL* have been associated with fetal growth (Eberlein et al., 2009) and calving ease in cattle (Druet et al., 2013). Plassais et al. (2019) detected a significant association with *LCORL* and height, weight, and life span in canids. Accordingly, *NCAPG* and *LCORL* genes have agricultural and adaptive importance and have been associated with allelic heterogeneity in selection signatures (Rochus et al., 2018).

Naval-Sanchez et al. (2018) analyzed 43 sheep breeds and 17 Asiatic mouflon with genome sequence resolution. On OAR6, between 37.42 and 37.51 Mb, they found a signature of selection, and the gene associated with this position was *LCORL*, which has a function related to weight and height (as the *NCAPG* gene). These authors indicated that domestic sheep contain a selective sweep in the *LCORL* promoter region, whereas mouflon contain a sweep downstream of *NCAPG*.

To conclude, ROH and single marker F_ST_ analyses agreed that selection signatures exist around markers *OAR6_41583796.1* and *OAR6_41709987.1* that were located near the *NCAPG* and *LCORL* genes. One factor involved in shaping ROH patterns in French Dairy sheep was selection. Accordingly, the preliminary identification of ROH can suggest the presence of selection. However, for the identification of potential candidate genes, ROH detection should be combined with other approaches to improve mapping accuracy.
